# Climate Change and the Potential Spreading of Marine Mucilage and Microbial Pathogens in the Mediterranean Sea

**DOI:** 10.1371/journal.pone.0007006

**Published:** 2009-09-16

**Authors:** Roberto Danovaro, Serena Fonda Umani, Antonio Pusceddu

**Affiliations:** 1 Department of Marine Sciences, Polytechnic University of Marche, Ancona, Italy; 2 Department of Life Sciences, University of Trieste, Trieste, Italy; Mt. Alison University, Canada

## Abstract

**Background:**

Marine snow (small amorphous aggregates with colloidal properties) is present in all oceans of the world. Surface water warming and the consequent increase of water column stability can favour the coalescence of marine snow into marine mucilage, large marine aggregates representing an ephemeral and extreme habitat. Marine mucilage characterize aquatic systems with altered environmental conditions.

**Methodology/Principal Findings:**

We investigated, by means of molecular techniques, viruses and prokaryotes within the mucilage and in surrounding seawater to examine the potential of mucilage to host new microbial diversity and/or spread marine diseases. We found that marine mucilage contained a large and unexpectedly exclusive microbial biodiversity and hosted pathogenic species that were absent in surrounding seawater. We also investigated the relationship between climate change and the frequency of mucilage in the Mediterranean Sea over the last 200 years and found that the number of mucilage outbreaks increased almost exponentially in the last 20 years. The increasing frequency of mucilage outbreaks is closely associated with the temperature anomalies.

**Conclusions/Significance:**

We conclude that the spreading of mucilage in the Mediterranean Sea is linked to climate-driven sea surface warming. The mucilage can act as a controlling factor of microbial diversity across wide oceanic regions and could have the potential to act as a carrier of specific microorganisms, thereby increasing the spread of pathogenic bacteria.

## Introduction

**Figure 1:**
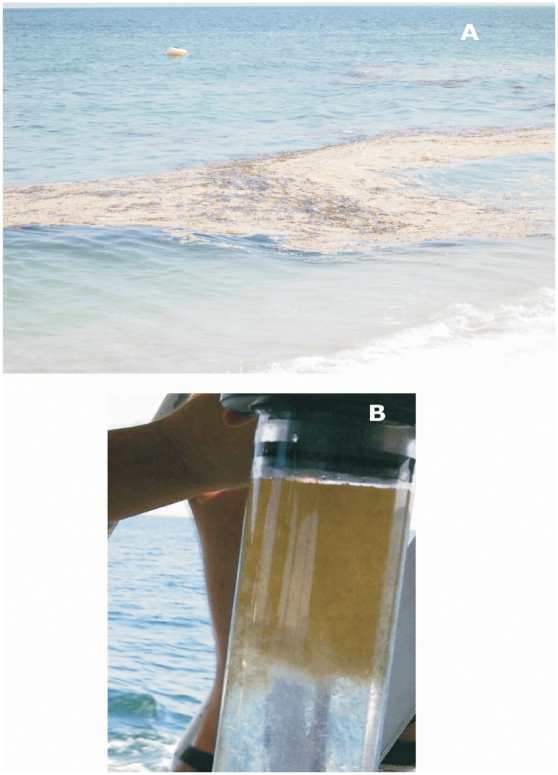
Mucilage and the “mare sporco” (dirty sea) phenomenon. Image of mucilage in surface off-shore waters (A) and in an advanced stage of coalescence, after sampling (B).

Marine snow (i.e., amorphous aggregates with a size ranging from a few millimetres to several metres) is ubiquitous in the oceans of the World [1]. Water column stratification under summer conditions favours the progressive coalescence of small-sized aggregates into large massive sheets, thin layers, flocs and clouds, which are collectively known as marine mucilage [2]. Mucilage is a gelatinous evolving stage of marine snow (Figure 1), which can reach huge dimensions and cover areas of hundreds of kilometres of coastline [3]–[6].

Mucilage is made of exopolymeric compounds with highly colloidal properties that are released by marine organisms [7] through different processes, including phytoplankton exudation of photosyntetically-derived carbohydrates produced under stressful conditions [8], [9] (e.g., P-limited diatoms that produce large amounts of polysaccharides [10]–[14]) and through death and decomposition of cell-wall debris [15], [16]. Such a release can be coupled with a limited ability of prokaryotes to hydrolyze these exopolymers by means of extracellular enzymes [17]–[18] leading to the release and accumulation of large molecular weight compounds in the system [19]–[26]. These processes can be associated with viral infections of prokaryotes and phytoplankton and the consequent cell lysis (viral shunt), which further contributes to release and accumulation of dissolved organic matter in the water column [27]–[31]. Whatever the causes triggering the formation of marine mucilage, this phenomenon has created increasing concern in coastal areas due to its socio-economical consequences.

Worldwide the highly productive and shallow Adriatic Sea (and particularly its Northern portion) within the Mediterranean basin is the area most severely affected by the outbreak of massive marine mucilage. Mucilage was reported here for the first time in 1729, and was originally described as a “dirty sea” phenomenon (“mare sporco”) because it causes the clogging of fishing nets [32]. Since then, the presence of mucilage has been reported sporadically, but in the last three decades, the frequency of this phenomenon appears to have increased considerably [2]. The presence of mucilage makes the seawater unsuitable for bathing because of the bad smell produced, and the adherence of the mucilage on the skin of bathers. Marine mucilage floating on the surface or in the water column can display a long life span (up to 2–3 months) and once settled on the sea bottom, these large aggregates coat the sediments, extending in certain cases for kms and causing hypoxic and/or anoxic conditions [2]. The consequent suffocation of benthic organisms (including bottom-associated nekton) [24] provokes serious economical damage to tourism and fisheries [33].

In the present study we hypothesized that: i) marine mucilage can represent a new, though ephemeral, substrate for microbial colonization, including pathogenic forms, ii) increasing frequency of mucilage outbreaks in the Mediterranean Sea is linked to recent climate change and iii) increasing occurrence of marine mucilage can increase spreading of marine diseases.

## Results

### Viruses and prokaryotes associated with marine mucilage

**Figure 2:**
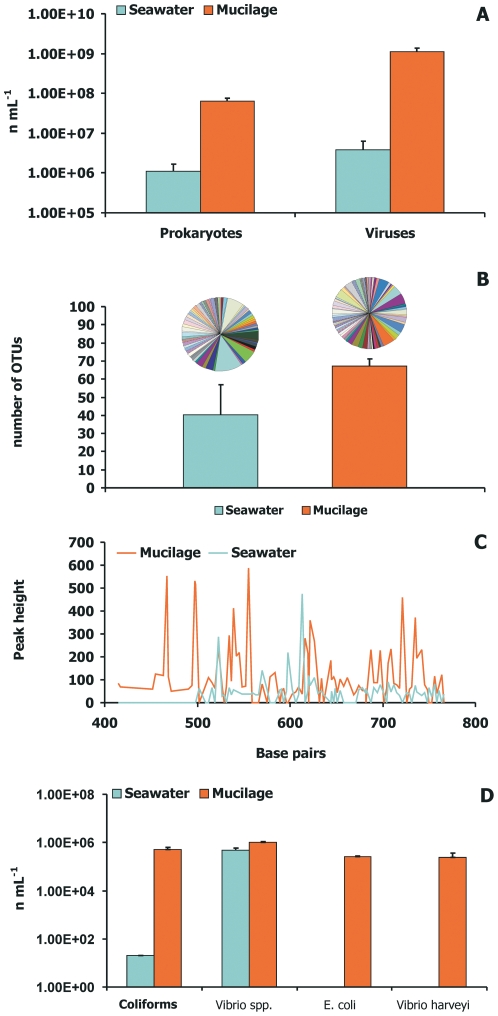
Microbial abundance and diversity in marine mucilage and surrounding seawater. (A) viral and prokaryotic abundance; (B) bacterial diversity (as OTU, Operational Taxonomic Units); (C) electropherograms of DNA extracted from a mucilage sample and surrounding seawater; (D) number of pathogens. Panel B illustrates diversity of bacterial assemblage: species in common are represented using the same colour. In Panel C, each peak corresponds to an interval of ±2 base pairs, is assumed to represent a different OTU, and the height of the peak is assumed to represent the quantity of each single OTU.

The microscopic analyses we conducted on samples of marine mucilage revealed the presence of huge prokaryotic and viral abundances (on average 3.76±2.53 10^6^ cells and 1.11±0.26 10^9^ viruses mL^−1^). Prokaryotic and viral abundances in mucilage were significantly higher (ANOVA P<0.001) than in surrounding seawater (Figure 2A). Molecular analyses demonstrated the presence of a huge bacterial diversity within the mucilage, with a number of ribotypes significantly higher (approximately double; ANOVA P<0.01) than in surrounding seawater (Figure 2B and 2C). The number of bacterial taxa encountered in the mucilage matrix contributed for ca 68% to the total number of bacterial taxa identified. We found that more than 90% of the bacterial taxa encountered in the mucilage were not found in the surrounding seawater.

Molecular analyses based on fluorescent *in situ* hybridization revealed that mucilage contained a large number of pathogenic bacteria (Figure 2D). The abundance of coliforms per unit of volume in marine mucilage per unit of volume was four orders of magnitude higher than in surrounding seawater, and *Vibrio* spp. were significantly more abundant than in the water column (ANOVA, p<0.01). The use of molecular fingerprinting techniques (ARISA) carried out both on mucilage and on the surrounding seawater provided evidence that mucilage aggregates not only entrap prokaryotes present in the water column, but also contain bacterial species (*Escherichia coli* and *Vibrio harveyi*), which were absent in surrounding seawater.

### Frequency and distribution of mucilage outbreaks

**Figure 3:**
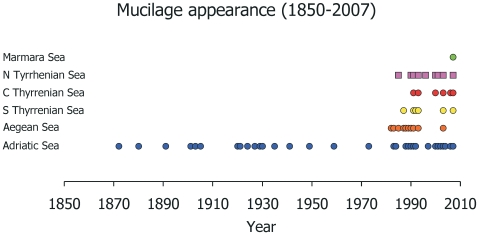
The areas in the Mediterranean Sea where the mucilage has been documented and the years of appearance.

Analysis of historical reports indicated that the frequency of the mucilage has increased almost exponentially in the last two decades in the Mediterranean Sea (Figure 3). Prior to 1920 mucilage events have been reported only in the Adriatic Sea. Since 1980 mucilage events were also reported from the Aegean, Northern and Tyrrhenian Seas. In the last 30 years the area with the highest number of mucilage outbreaks was the Adriatic Sea (n = 14) followed by the Tyrrhenian Sea (n = 11), and the Aegean Sea (n = 9).

The analysis of temperature anomalies over the last 60 years revealed that these have remained constantly positive since 1977 with values ranging from 0.097 (1986) to 0.524 (1998). Mucilage occurrence displayed a significant relationship (Spearman Rank correlation r_s_ = 0.50, P<0.003; n = 34) with climate change (as temperature anomalies, annual average; www.cpc.noaa.gov) in the last 60 years.

## Discussion

### Mucilage as potential carriers of marine diseases

Mucilage is able to entrap high abundances of a wide range of organisms from small phytoplankton to large zooplankton, and is capable of scavenging plankton and detrital particles suspended in the water column [34], [35]. Our results provide evidence that marine mucilage is also a major repository for prokaryotes and viruses, which displayed a concentration factor (as ratio between abundance in mucilage: abundance in seawater per unit of volume) ranging from 10^3^–10^4^. No other organisms entrapped in the mucilage were as enriched. The huge abundance of prokaryotes and viruses within the mucilage could be due to the ability of these large aggregates to entrap into free-living viruses and prokaryotes from the water column. In addition the mucilage represents a source of organic molecules potentially utilizable for sustain prokaryotic metabolism and growth. Typically viral and prokaryotic abundances in aquatic systems co-vary [23]. This also occurred in marine mucilage where a significant correlation between prokaryote and viral abundance was observed (Spearman-rank correlation, r_s_ = 0.99; p<0.001). The values of the ratio of virus-to-prokaryotic abundance in the mucilage (always >17) were significantly higher than values typically reported for seawater [36], suggesting that mucilage can be characterized by a strong viral shunt with a much higher probability of virus-host contact than in surrounding seawater [37]. Similar values have been recently reported for deep-benthic marine systems, where a viral shunt proved to be extremely important [38]. The high viral abundance can influence prokaryotic dynamics by killing the prokaryotes and causing the release of particulate and dissolved organic material, which can then contribute to the renewal and persistence of the mucilage [23].

The use of fingerprinting techniques indicated that marine mucilage is also a hot spot of bacterial diversity. The number of ribotypes in the mucilage was on average ca 65% higher than in the surrounding seawater. Since mucilage can remain suspended in the seawater for months, it is possible that several prokaryotic species are released in areas far from their origin during dispersal of mucilage by currents.

Most known diseases affecting both marine invertebrate and vertebrates, such as those referred to as yellow blotch, dark spots, and the so-called rapid tissue necrosis, are only described for their symptoms and have not been characterized by their aetiology or pathogenesis [39]. Detailed microbiological investigations of marine pathogens have been hampered previously by the difficulties in cultivating them. Only recently has the development of molecular methodologies allowed progress [40]. In the present study, the use of molecular techniques such as the fluorescence *in situ* hybridization (FISH) revealed the presence of a conspicuous number of pathogenic bacteria (e.g., *Vibrio harveyi*) in the mucilage, but not in surrounding seawater, which have the potential to infect a wide range of organisms [41].

FISH analyses revealed also the presence of very high abundances of total Coliforms and *E. coli*, which are common indicators for the potential presence of pathogens. The presence of these bacteria in the aggregates suggests that mucilage could have potentially negative consequences on human health. The abundance of these bacteria are typically monitored in coastal environments as they are indicators of water quality and their presence above certain threshold levels limits recreational activities at sea. The ability of mucilage to potentially concentrate high abundances of pathogenic bacteria is consistent with the appearance of dermatitis and other syndromes associated to human contact with the mucilage [42].

Although the mechanisms by which the mucilage hosts large numbers of pathogens are not entirely clear, it might be hypothesized that the complex organic matrix of the mucilage offers micro-niches to pathogens with favourable conditions for their colonization and survival [43]. Results reported here also suggest that if the expansion of the mucilage occurrence will continue in the future, it could be associated with increased outbreaks of diseases caused by the potential release of large numbers of pathogenic bacteria from the mucilage.

### Mucilage and climate change

The analysis of historical reports in the Mediterranean Sea indicates that the occurrence of mucilage events is increasing and spreading to several regions beyond the Adriatic Sea, where it was documented for the first time. Mucilage is not closely associated with the presence of eutrophic conditions, as several mucilage outbreaks have been recently observed in oligotrophic seas, such as the Aegean Sea. Moreover, the frequency of mucilage outbreaks in the Adriatic Sea has increased in the last 2 decades concurrently with a significant decrease in primary production. Furthermore there is no correlation between the number of mucilage records per decade and the decadal mean of Po river annual discharge in the Adriatic Sea (Spearman r_s_ = −0.333; not significant, data not shown). It therefore can be hypothesised that the expansion of mucilage outbreaks in different areas of the Mediterranean Sea (including coastal regions that have no previous records for this phenomenon, such as the Marmara Sea [44]) do not result from increased primary productivity of the system (i.e., eutrophication).

**Figure 4:**
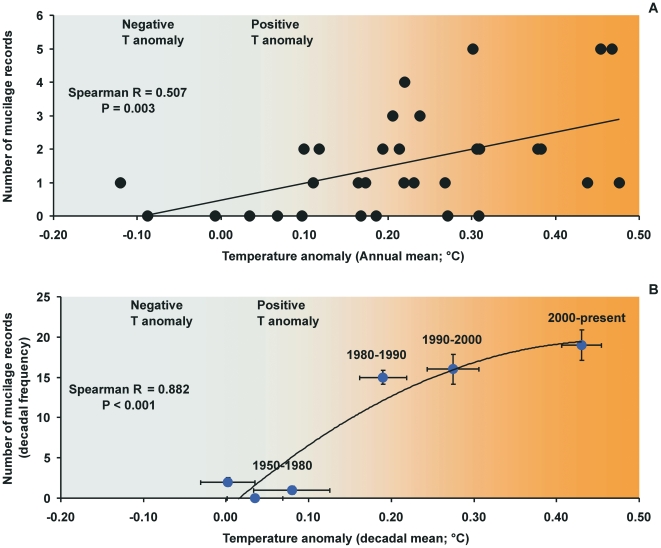
Relationships between mucilage occurrence in the Mediterranean Sea and climate change (as magnitude of the thermal anomalies) on (A) annual basis, (B) decadal basis. Annual and decadal temperature anomalies were retrieved from www.cpc.noaa.gov. Records of mucilage appearance in the Mediterranean Sea are detailed in Text S1.

Although recent studies have suggested that a climatic shift occurred in 1987 in the Mediterranean (e.g. the so called East Mediterranean Transient [45] could have been responsible for a shift in ecosystem functioning [46]), the linkage between mucilage occurrence and climate change has been almost completely neglected. Our analysis based on a record of approximately 60 years of mucilage appearance in the Mediterranean Sea (1950–2008) has revealed that patterns of climate anomalies (e.g., the positive anomaly of the surface temperature) explained a large proportion of variance in mucilage outbreaks, on an annual and decadal basis (Figure 4).

The hypothesis of a link between mucilage formation and climate-driven temperature anomalies is suggested by the apparent progressive extension of the duration of this phenomenon. In the Adriatic Sea, for instance, marine aggregates generally appear from May to July and evolve into mucilage through the Summer (with a peak in August). Recently (i.e., in 2003, 2006, 2007 and 2008), mucilage has appeared much earlier (first recorded in November/December and January, depending upon the area). The Winter 2006–2007 was the warmest over the last 30 years, with average temperatures up to 2–3°C above the previous mean temperatures (http://www.noaanews.noaa.gov/stories2007/s2798.htm), and the spatial extent and persistence of mucilage reached unprecedented levels. In March 2007, for instance, marine mucilage was seen to stretch along more than 2,500 km of the Italian coastline. Massive aggregates lasted, almost continuously, for more than five months. The last outbreaks have been reported in autumn of 2008. This tendency, however, needs to be monitored in the future for consistency.

The presence of temperature anomalies alone cannot be used to predict the occurrence of mucilage phenomenon on a basin scale because several other (local) factors may promote the mucilage formation and/or increases in the magnitude of this phenomenon including the hydrodynamic regime (i.e., current speed and water mass turnover), oxygen availability, and other factors. However, the link between climate anomalies and the occurrence of mucilage is evident and, in the light of the warming trend of the Mediterranean Sea [47], the mucilage phenomenon could increase in the future.

The coastal areas of the Mediterranean Sea that are repeatedly affected by mucilage appearance share many common environmental problems. Most of these systems have a long history of human exploitation [48], [49], including over-fishing (which through trawling can be also responsible for the alteration of the benthic biogeochemical cycles) [50], presence of macro- and micro-pollutants (which can have a strong impact on microbial-loop functioning and cause the increase of viral infection [51] or the outbreak of microbial diseases [52] and altered ecosystem functioning. Perhaps the misuse of these coastal environments might exacerbate this phenomenon. Mucilage, in turn, can induce hypoxic phenomena and even promote extensive anoxia [53] resulting in a decreased production of “ecosystem goods and services”[54], and a lower ecosystem resiliency (ability to recover after adverse impacts).

If the mucilage phenomenon continuous to increase in frequency and duration, and to spread around the coastal areas of the Mediterranean Sea, an increased frequency and extension of some marine diseases may result with potential consequences to human health [55]. In the past, the “*Vibrio cholerae* paradigm” has represented the first important example of the cascade effects of climate change on human health [56]. *Vibrio cholera*, indeed, lives attached to the exoskeleton of marine copepods, which depend on phytoplankton blooms for their nutrition. These in turn are influenced by climate change [56]. We propose mucilage as a potentially novel paradigm of the ecosystem alteration caused by the synergistic effect of climate change and misuse of marine resources. Mucilage on one hand represents symptomatic response of the marine ecosystem to direct and indirect anthropogenic impacts, and on the other a potentially expanding carrier of viruses and bacteria, including pathogenic forms that are harmful for the health of humans and marine organisms.

## Methods

### Sampling

Samples of seawater and mucilage were collected from coastal waters of the Adriatic Sea in 2007 by means of sterilized Niskin bottles and by 100 mL syringes operate by SCUBA divers, respectively. Fifty mL each of mucilage and seawater were immediately stored at −20°C for subsequent viral and prokaryotic counts (within 48 h). Additional water samples were filtered on 0.2-µm pore-size polycarbonate filters and immediately processed together with fresh mucilage samples for DNA extraction.

### Viral and prokaryotic abundance and biodiversity in seawater and mucilage

Viral and prokaryotic abundances were determined by epifluorescence microscopy after staining with SYBR Green I and normalised to mL of seawater or mucilage [37].

Automated rRNA Intergenic Spacer Analysis (ARISA) was carried out on seawater and mucilage samples. DNA was extracted from mucilage samples (ca. 1 mL) by means of the UltraClean Soil DNA Isolation kit (MoBio Laboratoires Inc., California, USA) [57]. Extracted DNA was determined fluorometrically using SYBR Green I (Molecular Probes, USA) and quantified using standard solutions of genomic DNA from *E. coli* [58]. The DNA was then amplified using universal bacterial primers 16S-1392F (5′-GYACACACCGCCCGT-3′) and 23S-125R (5′-GGGTTBCCCCATTCRG-3′), which amplify the bacterial ITS1 region in the rRNA operon plus ca. 282 bases of the 16S and 23S rRNA. The reverse primer 23S-125R was fluorescently labelled with the fluorochrome HEX (MWGspa BIOTECH). PCR reactions were performed in 50-µl volumes in a thermalcycler (Biometra, Germany) using the MasterTaq® kit (Eppendorf AG, Germany). 30 PCR-cycles were used, consisting of 94°C for 1 minute, 55°C for 1 minute and 72°C for 2 minute, preceded by 3 minutes of denaturation at 94°C, followed by a final extension of 10 minutes at 72°C. To check for eventual contamination of the PCR reagents, negative controls containing the PCR-reaction mixture but without the DNA template were run during each PCR analysis. Positive controls, containing genomic DNA of *Escherichia coli*, were also used. PCR-products were checked on agarose-TBE gel (1%), containing ethidium bromide for DNA staining and visualization. For each mucilage sample, two different PCR reactions were run and then pooled together to minimize stochastic PCR biases. This process was carried out in duplicate, for a total of 4 different PCR reactions for each sample. The two resulting PCR combined products were then purified using the Wizard PCR clean-up system (Promega, Wisconsin, USA), resuspended in 50 µl of milliQ water supplied with the clean-up system and then quantified spectrofluorimetrically as described above. For each ARISA analysis, about 5 ng of amplicons were mixed with 14 µl of internal size standard (GS2500-ROX; Applied Biosystems, Foster City, Calif.) in deionized formamide, then denatured at 94°C for 2 minutes and immediately chilled in ice. Automated detection of ARISA fragments was carried out using an ABI Prism 3100 Genetic Analyzer (Applied Biosystems). ARISA fragments in the range 390–1400 bp were determined using Genescan analytical software 2.02 (ABI). Bacterial phylotype/genotype richness was expressed as the total number of peaks within each electropherogram.

### Identification of pathogens by Fluorescent *In Situ* Hybridization (FISH)

For the detection and enumeration of potentially pathogenic microorganisms (i.e. total Coliforms, *Escherichia coli*, *Vibrio* spp. and *Vibrio harveyi*) we used the FISH (Fluorescent *In Situ* Hybridization) technique targeting the bacterial 16S rRNA. From each sample, ca. 1 mL of each mucilage sample was fixed in replicate in a 1∶1 sterile solution of PBS:ethanol and sonicated three times. Aliquots of each sample were then properly diluted and filtered through 0.2 µm black polycarbonate filters (Nuclepore). The filters were then hybridized using Cy3-labeled probes under appropriate hybridization condition for each probe and washed using appropriate washing buffers. Then, each filter was counterstained with DAPI (0.5 µg mL^−1^), mounted onto microscopic slides and observed under epifluorescence microscopy. We used the probes “D”, *Colinsitu, GV, VH-2* for counting Total Coliforms, *Escherichia coli*, *Vibrio* spp., and *Vibrio harveyi*, respectively.

### Historical data collection and statistical analyses

For the purposes of this research, we searched for mucilage and macro-aggregate (>500 µm in size) records in oceans worldwide in published literature (Sciencedirect, ASFA). The research for mucilage records was also extended to include the whole of the Internet, to identify events not documented in scientific papers (Table S1). Annual mean data on Po river discharge (1990–2007) in the Adriatic Sea have been extracted from the Hydrological Annales of the Regional Agency for Prevention and Climate of the Emilia Romagna Region (www.arpa.emr.it).

Quantitative differences in microbial variables between mucilage and seawater samples were tested using one-way analysis of variance (ANOVA). The type of substrate (mucilage vs. seawater) was treated as a fixed factor with two levels and significant differences were also assessed using a post-hoc Student-Newman-Kuels' test (SNK). ANOVA and SNK tests were carried out using the software GMAV 5.0 (University of Sidney, Australia). The relationships between microbial variables within mucilage samples and within seawater were tested using a Spearman-rank correlation analysis.

## Supporting Information

Table S1 List of the records of the appearance of mucilage in the Mediterranean Sea and specific geographic locations.
(0.08 MB DOC)

Text S1 This file contains all info collected on historical events of mucilage appearance in the Mediterranean Sea.
(0.07 MB DOC)
