# Supplementary material for: Climate Change and the Potential Spreading of Marine Mucilage and Microbial Pathogens in the Mediterranean Sea
Source: PLoS One. 2009 Sep 16;4(9):e7006. doi: 10.1371/journal.pone.0007006 (PMC2739426; doi:10.1371/journal.pone.0007006)
Supplement: Table S1 — List of the records of the appearance of mucilage in the Mediterranean Sea and specific geographic locations. (0.08 MB DOC) [file pone.0007006.s001.doc]

Table S1

| **Year** | **Region** | **Reference** |
| --- | --- | --- |
| 1729 | Adriatic Sea | [S4] |
| 1872 | Adriatic Sea | “ |
| 1880 | Adriatic Sea | “ |
| 1891 | Adriatic Sea | “ |
| 1903 | Adriatic Sea | “ |
| 1905 | Adriatic Sea | “ |
| 1920 | Adriatic Sea | “ |
| 1921 | Adriatic Sea | [S5] |
| 1922 | Adriatic Sea | “ |
| 1924 | Adriatic Sea | “ |
| 1927 | Adriatic Sea | “ |
| 1929 | Adriatic Sea | [S4] |
| 1930 | Adriatic Sea | “ |
| 1935 | Adriatic Sea | [S5] |
| 1941 | Adriatic Sea | “ |
| 1949 | Adriatic Sea | [S6] |
| 1951 | Adriatic Sea | “ |
| 1959 | Adriatic Sea | [S5] |
| 1973 | Adriatic Sea | “ |
| 1976 | Adriatic Sea (Quarner) | [S7] |
| 1983 | Adriatic Sea (Quarner) | “ |
| 1988 | Adriatic Sea | [S8] |
| 1989 | Adriatic Sea | “ |
| 1990 | Adriatic Sea (Rijeka Bay) | “ |
| 1991 | Adriatic Sea | [S6] |
| 1997 | Adriatic Sea | “ |
| 2000 | Adriatic Sea | [S9] |
| 2001 | Adriatic Sea | “ |
| 2002 | Adriatic Sea | “ |
| 2003 | Adriatic Sea | Media report |
| 2004 | Adriatic Sea | Media report |
| 2007 | Adriatic Sea | Media report |
| 2002 | Adriatic Sea | [S10] * |
| 1985 | Northern Tyrrhenian Sea | [S11] * |
| 1989 | Northern Tyrrhenian Sea | [S12] * |
| 1991 | Northern Tyrrhenian Sea | [S13] * |
| 1991 | Northern Tyrrhenian Sea | [S14] * |
| 1996 | Northern Tyrrhenian Sea | [S15] * |
| 1999 | Northern Tyrrhenian Sea | [S16] * |
| 2000 | Northern Tyrrhenian Sea | [S17] * |
| 2001 | Northern Tyrrhenian Sea | “ |
| 2003 | Northern Tyrrhenian Sea | [S18] * |
| 2007 | Northern Tyrrhenian Sea | Media report |
| 1987 | Southern Tyrrhenian Sea | [S11] * |
| 1990 | Southern Tyrrhenian Sea | [S11] |
| 1991 | Southern Tyrrhenian Sea | [S19] |
| 1992 | Southern Tyrrhenian Sea | “ |
| 1993 | Southern Tyrrhenian Sea | [S20] |
| 2003 | Southern Tyrrhenian Sea | Media report |
| 2007 | Southern Tyrrhenian Sea | Media report |
| 1991 | Central Tyrrhenian Sea | [S11] |
| 1993 | Central Tyrrhenian Sea | [S21] * |
| 2000 | Central Tyrrhenian Sea | [S22] * |
| 2006 | Central Tyrrhenian Sea | Media report |
| 2007 | Central Tyrrhenian Sea | Media report |
| 1982 | Aegean Sea | [S23] |
| 1983 | Aegean Sea | “ |
| 1985 | Aegean Sea | “ |
| 1987 | Aegean Sea | “ |
| 1988 | Aegean Sea | “ |
| 1989 | Aegean Sea | “ |
| 1990 | Aegean Sea | “ |
| 1991 | Aegean Sea | “ |
| 1993 | Aegean Sea | “ |
| 2003 | Aegean Sea | [S24] |
| 2007 | Alboran Sea-Gibraltar | S. Fonda Umani (pers. obs.) |
| 2008 | Marmara Sea | M. Isinibilir (pers. comm.) |
| 2007-2008 | Marmara Sea | [S25] |
