# Supplementary material for: Climate Change and the Potential Spreading of Marine Mucilage and Microbial Pathogens in the Mediterranean Sea
Source: PLoS One. 2009 Sep 16;4(9):e7006. doi: 10.1371/journal.pone.0007006 (PMC2739426; doi:10.1371/journal.pone.0007006)
Supplement: Text S1 — This file contains all info collected on historical events of mucilage appearance in the Mediterranean Sea. (0.07 MB DOC) [file pone.0007006.s002.doc]

Supporting Information

**Climate Change and the spreading of marine mucilage and microbial pathogens in the Mediterranean Sea**

**Roberto Danovaro^1,*^, Serena Fonda Umani^2^, Antonio Pusceddu^1^**

^1^ Department of Marine Science, Polytechnic University of Marche, Ancona, Italy

^2^ Department of Life Sciences, University of Trieste, Trieste, Italy

**^🖃^ Corrisponding author -** E-mail: [r.danovaro@univpm.it](mailto:r.danovaro@univpm.it)

**Data Sources**

Mucilage aggregates are considered to be an exacerbated and evolving stage of "marine snow" [S1-S3] that appears under environmental conditions that favour the progressive coalescence of marine macro-aggregates, such as during the Summer water column stratification. For the purposes of this overview, we searched for mucilage records in the Mediterranean Sea in published literature (Sciencedirect, ASFA, Scholar Google, Scopus). The research for mucilage records was also extended to include the whole of the Internet, to identify events not documented in scientific papers. The entire list of the records found is detailed in Supplemental Table S1.

**Supplemental References**

1. Herndl GJ, Peduzzi P (1988) Ecology of amorphous aggregations (marine snow) in the Northern Adriatic sea: I general considerations. *PSZNI Marine Ecology* 9: 79-90.
2. Herndl GJ (1992) Marine snow in northern Adriatic Sea: possible causes and consequences for a shallow ecosystem. *Marine Microbial Food Webs* 6: 149-172.
3. Fogg GE (1995) Some speculations on the nature of the pelagic mucilage community of the northern Adriatic Sea. Science of the Total Environment 165: 59-63.
4. Fonda Umani S, Ghirardelli E, Specchi M (1989) Gli episodi di "mare sporco" nell'Adriatico dal 1729 ai giorni nostri. Regione Autonoma Friuli-Venezia Giulia. Direzione Regione Ambiente (ed) pp. 178.
5. Deserti M, Cacciamani C, Chiggiato J, Rinaldi A, Ferrari CR (2005) Relationships between northern Adriatic Sea mucilage events and climate variability. Science of the Total Environment 353: 82-88.
6. Sellner KG, Fonda Umani S (1999) Dinoflagellate Blooms and Mucilage Production. In: (Malone TC, Malej A, Harding LWJr, Smodlaka N, Turner RE, eds) Chapter 6. Coastal and Eustarine Studies 55: 173-206.
7. Pucher-Petkovic T, Marasovic I (1987) Contribution a la Connaissance d'une Pousee Extraordinaire d'Algues Unicellulaires (Adriatique Septentrionale), CENTRO 1: 33-44.
8. Fonda Umani S, Franco P, Ghirardelli E, Malej A (1992) Outline of oceanography and the plankton of the Adriatic Sea. In: Marine Eutrophication and Population Dynamics (Colombo G, Ferrari I, Ceccherelli VU, Rossi R, eds*)* Proceedings of the 25th EMBS: 347-365.
9. Precali R, Giani M, Marini M, Grilli F, Ferrari CR et al. (2005) Mucilaginous aggregates in the Northern Adriatic in the period 1999-2002: typology and distribution. Science of the Total Environment 353: 10-23.
10. Sartoni G, Urbani R, Sist P, Berto D, Nuccio C et al. (2008) Benthic mucilaginous aggregates in the Mediterranean Sea: Origin: chemical composition and polysaccharide characteizaion. Marine Chemistry 111: 184- 198.
11. Innamorati M (1995) Hyperproduction of mucilages by micro and macro algae in the Tyrrhenian Sea. Science of the Total Environment 165: 65-81.
12. Hoffmann L, Billard C, Janssens M, Leruth M, Demoulin V (2000) Mass Development of Marine Benthic Sarcinochrysidales (Chrysophyceae s.l.) in Corsica. Botanica Marina 43: 223-232.
13. Innamorati M, Raddi E, Buzzichelli S, Melley A, Balzi M (1992) Mucilages in the Tyrrhenian Sea. Proceedings of the 23^rd^ Congress of the Italian Society of Marine Biology 6: 23-26.
14. Sartoni G, Sonni C (1992) *Trybonema marinum* J. Feldmann e *Acinetospora crinita* (Carmichael) Sauvageau nelle formazioni mucillagginose bentoniche osservate sulle coste toscane nell’estate 1991. Informatore Botanico Italiano 23: 23-30.
15. Janssen M (1996) Filamentous and mucilaginous algal blooms in a Corsican Bay (Calvi-France) Harmful Algal News 7 pp.
16. De Philippis R, Sili C, Faraoni C, Vincenzini M (2002) Occurence and significance of exopolysaccharide-producing cyanobacteria in the benthic mucilaginous aggregates of the Tyrrhenian Sea (Tuscan Arcipelago). *Annales of Microbiology* 52: 1-11.
17. Giuliani S, Virno Lamberti C, Sonni C, Pellegrini D (2005) Mucilage impact on gorgonians in the Tyrrhenian Sea. Science of the Total Environment 353: 340-349.
18. Schiaparelli S, Castellano M, Povero P, Sartoni G, Cattaneo R (2007). A benthic mucilage event in North-Western Mediterranean Sea and its possible relationships with the summer 2003 European heatwave: short term effects on littoral rocky assemblages. Marine Ecology 28: 1-13.
19. Calvo S, Barone R, Naselli Flores L (1995) Observations on mucus aggregates along Siciliancoasts durino 1991-1992. Science of the Total Environment 165: 23-31.
20. Mistri M, Ceccherelli VU (1996) Effects of a mucilage event on the Mediterranean gorgonian *Paramuricea clavata*. 1. Short-term impacts at the population and colony levels. Italian Journal of Zoology 63: 221-230.
21. Olianas A, Fadda MB, Boffi A, Murenu M, Deiana A et al. (1996) Benthic mucilagigenous aggregates:biochemical characterization and ligand binding properties. Marine Environmental Research 41: 1-14.
22. Lorenti M, Buia MC, Di Martino V, Modigh M (2005) Occurence of mucous aggregates and their impact on Posidonia oceanica beds. Science of the Total Environment 353: 369-379.
23. Gotsis-Skretas O (1995) Mucilage appearances in Greek waters during 1982-1994. Science of the Total Environment 165: 229-230.
24. Scoullos M, Plavšić M, Karavoltsos S, Sakellari A (2006) Partitioning and distribution of dissolved copper, cadmium and organic matter in Mediterranean marine coastal areas: The case of a mucilage event. Estuarine Coastal and Shelf Science 67: 484-490.
25. Aktan Y, Dede A, Ciftci PS (2008) Mucilage event associated with diatoms and dinoflagellates in Sea of Marmara, Turkey. Harmful Algae News, 36: 1- 3.
